# Supplementary material for: Splice-Junction-Based Mapping of Alternative Isoforms in the Human Proteome
Source: Cell Rep. Author manuscript; Available in PMC 2020 Jan 15. (PMC6961840; doi:10.1016/j.celrep.2019.11.026)
Supplement: 3 [file NIHMS1546469-supplement-3.zip › DF2/PXD006675/RightAtrium-376-O75071-ETPGSNQIIPSPSATSELDNKTHSENLKQMGDR.pdf]

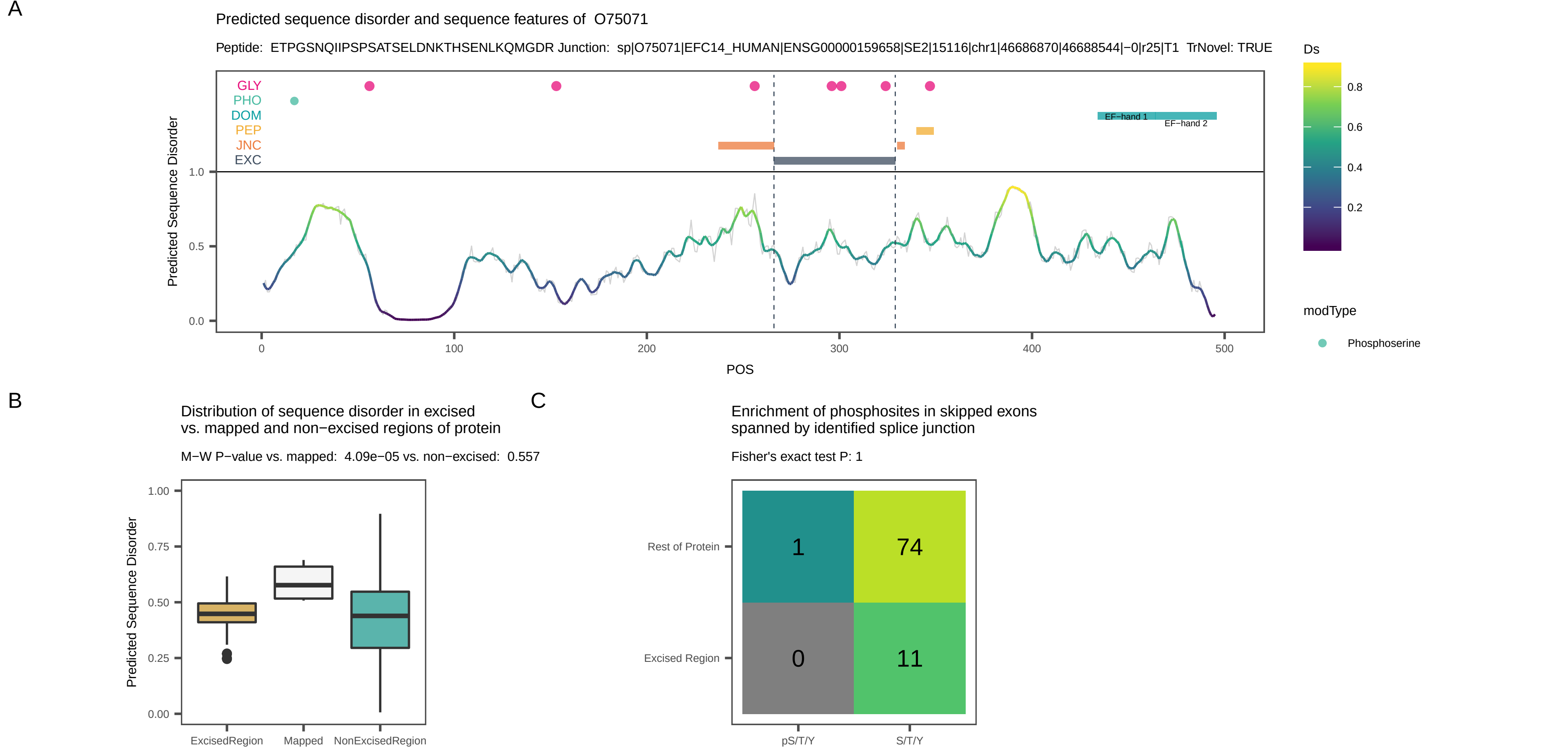
A
Predicted sequence disorder and sequence features of O75071
Peptide: ETPGSNQIIPSPSATSELDNKTHSENLKQMGDR Junction: sp|O75071|EFC14_HUMAN|ENSG00000159658|SE2|15116|chr1|46686870|46688544|−0|r25|T1 TrNovel: TRUE
GLY
PHO
DOM
PEP
JNC
EXC
EF−hand 1
EF−hand 2
Predicted Sequence Disorder
1.0
0.5
0.0
0
100
200
300
400
500
POS
Ds
0.8
0.6
0.4
0.2
modType
Phosphoserine
B
Distribution of sequence disorder in excised
vs. mapped and non−excised regions of protein
M−W P−value vs. mapped: 4.09e−05 vs. non−excised: 0.557
Predicted Sequence Disorder
1.00
0.75
0.50
0.25
0.00
ExcisedRegion
Mapped
NonExcisedRegion
C
Enrichment of phosphosites in skipped exons
spanned by identified splice junction
Fisher's exact test P: 1
Rest of Protein
Excised Region
1
74
0
11
pS/T/Y
S/T/Y
